# Long-Term Effects of Maternal Citrulline Supplementation on Renal Transcriptome Prevention of Nitric Oxide Depletion-Related Programmed Hypertension: The Impact of Gene-Nutrient Interactions

**DOI:** 10.3390/ijms151223255

**Published:** 2014-12-15

**Authors:** You-Lin Tain, Chien-Te Lee, Li-Tung Huang

**Affiliations:** 1 Department of Pediatrics, Kaohsiung Chang Gung Memorial Hospital and Chang Gung University College of Medicine, Kaohsiung 833, Taiwan; E-Mail: litung.huang@gmail.com; 2 Center for Translational Research in Biomedical Sciences, Kaohsiung Chang Gung Memorial Hospital and Chang Gung University College of Medicine, Kaohsiung 833, Taiwan; 3 Division of Nephrology, Departments of Medicine, Kaohsiung Chang Gung Memorial Hospital and Chang Gung University College of Medicine, Kaohsiung 833, Taiwan; E-Mail: ctlee33@adm.cgmh.org.tw; 4 Department of Traditional Chinese Medicine, Chang Gung University, Linkow 244, Taiwan

**Keywords:** citrulline, developmental programming, epigenetic regulation, hypertension, next generation sequencing, nitric oxide

## Abstract

Maternal malnutrition can elicit gene expression leading to fetal programming. l-citrulline (CIT) can be converted to l-arginine to generate nitric oxide (NO). We examined whether maternal CIT supplementation can prevent N^G^-nitro-l-arginine-methyl ester (l-NAME, NO synthase inhibitor)-induced programmed hypertension and examined their effects on the renal transcriptome in male offspring using next generation RNA sequencing (RNA-Seq) technology. Pregnant Sprague-Dawley rats received l-NAME administration at 60mg/kg/day subcutaneously via osmotic minipump during pregnancy alone or with additional 0.25% l-citrulline solution in drinking water during the whole period of pregnancy and lactation. Male offspring were assigned to three groups: control, l-NAME, and l-NAME + CIT. l-NAME exposure induced hypertension in the 12-week-old offspring, which CIT therapy prevented. Identified differentially expressed genes in l-NAME and CIT-treated offspring kidneys, including *Guca2b*, *Hmox1*, *Hba2*, *Hba-a2*, *Dusp1*, and *Serpine1* are related to regulation of blood pressure (BP) and oxidative stress. In conclusion, our data suggests that the beneficial effects of CIT supplementation are attributed to alterations in expression levels of genes related to BP control and oxidative stress. Our results suggest that early nutritional intervention by CIT has long-term impact on the renal transcriptome to prevent NO depletion-related programmed hypertension. However, our RNA-Seq results might be a secondary phenomenon. The implications of epigenetic regulation at an early stage of programming deserve further clarification.

## 1. Introduction

Nutrition in pregnancy and lactation can provoke long-term effects on the health of offspring. Maternal malnutrition impairs development via epigenetic alterations in genes involved in organogenesis, morphological changes, and adaptive physiological responses—a phenomenon referred to as developmental programming [1,2]. The most common phenotype in adult offspring is programmed hypertension [2,3]. We recently observed that programmed hypertension developed in the male offspring of pregnant rats exposed to nitric oxide (NO) deficiency, which was prevented by maternal l-citrulline supplementation [4].

l-citrulline is the endogenous precursor of l-arginine, the substrate for nitric oxide synthase (NOS) to generate NO. The amount of l-citrulline in the diet is negligible with the exception of watermelon and other related fruits. Experimental and clinical studies indicate that l-citrulline derived NO and polyamines are essential for embryonic survival, organogenesis, and fetal growth, as well as maintenance of vascular tone and hemodynamics [5]. l-citrulline is more effective than l-arginine to augment NO as it can bypass hepatic metabolism, it is well tolerated, and it is not metabolized by arginase [6]. Given that NO deficiency contributes to the development of hypertension [7], supplemental administration of l-citrulline is emerging as a promising treatment of many cardiovascular diseases with l-citrulline-NO deficiency, including hypertension [8]. In addition to hypertension, our recent reports demonstrated that l-citrulline therapy can restore NO bioavailability to prevent the transition of prehypertension to hypertension in spontaneously hypertensive rats [9] and the development of hypertension in different programming models [10,11,12].

The kidney is particularly susceptible to the insults of programming during nephrogenesis and has been identified as a key player in programmed hypertension [13]. Nutritional epigenetics play a critical role during placental maturation, organogenesis, and development [14]. Also, epigenetic regulation has been proposed to interpret the programming of hypertension [2,3]. Although nutrition during gestation plays a crucial role on gene expression, the potential role of l-citrulline on the renal transcriptome to prevent programmed hypertension remains unclear. In this study, we intended to examine whether l-citrulline can prevent NO deficiency-induced programmed hypertension in the offspring exposed to maternal N^G^-nitro-l-arginine-methyl ester (l-NAME, an NOS inhibitor) administration and to quantify the abundance of RNA transcripts in the offspring kidney using RNA next-generation sequencing (RNA-Seq).

## 2. Results

### 2.1. Animal Response to l-NAME and CIT

Litter sizes were not significantly altered by l-NAME or l-citrulline (CIT) exposure of the maternal rat (pups per litter: control = 11.6 ± 0.6, l-NAME = 11 ± 1, l-NAME + CIT = 11 ± 0.9). Male pup mortality rates, body weight (BW), kidney weight, and heart weight were not different among the three groups (Table 1). The systolic BP and mean arterial pressure of the l-NAME group were significantly higher than those in the control, which maternal CIT therapy prevented. Note that weights and BP data from three-month-old offspring, which in part was published previously [4], is included in this study for the sake of comparison.

**Table 1:** Weights and functional parameters in rats at three months of age.

Groups	Control	l-NAME	l-NAME + CIT
Mortality	0%	0%	0%
Body weight (BW) (g)	524 ± 30	478 ± 15	469 ± 9
Left kidney weight (g)	2.08 ± 0.16	1.87 ± 0.10	1.95 ± 0.04
Left kidney weight/100 g BW	0.39 ± 0.01	0.39 ± 0.01	0.42 ± 0.01
Heart weight (g)	1.56 ± 0.10	1.61 ± 0.04	1.62 ± 0.04
Heart weight/100 g BW	0.30 ± 0.01	0.34 ± 0.02	0.35 ± 0.01
Systolic blood pressure (mmHg)	149 ± 2	165 ± 1 ^a^	152 ± 2 ^b^
Diastolic blood pressure (mmHg)	102 ± 4	107 ± 4	87 ± 2 ^a,b^
Mean arterial pressure (mmHg)	117 ± 2	126 ± 3 ^a^	109 ± 1 ^b^

*N* = 6/group, ^a^ *p* < 0.05 *vs.* control; ^b^ *p* <0.05 *vs.* l-NAME.

### 2.2. The Effects of l-NAME and l-NAME + CIT on Renal Transcriptome

We next analyzed differential gene expression induced by l-NAME and l-NAME + CIT in 3-month-old offspring kidney. Among the differential expressed genes (DEGs), a total of 383 genes (198 up- and 185 down-regulated genes by l-NAME *vs.* control) met the selection criteria of (1) genes that changed by reads per kilobase of transcript per million mapped reads (RPKM) >0.3 and (2) minimum of 2-fold difference in normalized read counts between groups. *p* value was estimated for each gene and corrected for multiple testing (*q* value) by the Benjamini-Hochberg correction. The log2 fold change (FC) was used to partition the genes into up- and down-regulated groups. Next, a total of 337 DEGs (148 up- and 189 down-regulated genes) was noted in response to l-NAME + CIT therapy at 12 weeks of age.

**Figure 1:**
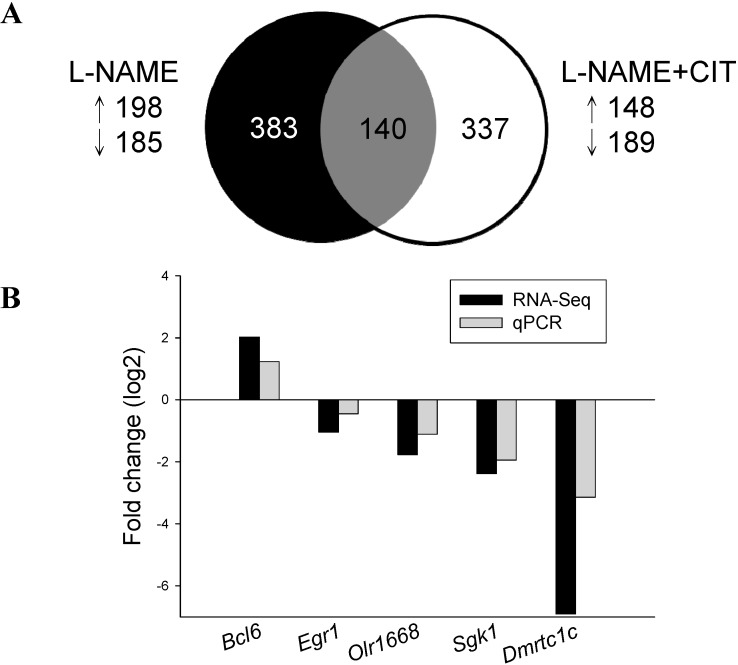
(**A**) Venn diagram depicting unique and shared (over-lapping circles) sets of differentially expressed genes (DEGs) in the kidney between maternal l-NAME therapy (black circle) and l-NAME plus l-citrulline therapy (white circle) at 12 weeks of age; (**B**) Confirmatory analysis of kidney gene expression by qPCR. Individual gene expression was determined by qPCR and expressed as fold change in log base 2 *vs.* control and graphed alongside fold change derived from RNA-Seq analysis.

Genes shared by two different groups are represented graphically by the Venn diagram shown in Figure 1A. Among them, 140 shared genes were identified. We validated five renal transcripts predicted to change significantly in the RNA-Seq dataset by quantitative real-time polymerase chain reaction (qPCR). The analysis confirmed all predicted transcriptional changes (Figure 1B).

We also found 9 significantly related Kyoto Encyclopedia of Genes and Genomes (KEGG) pathways in the kidney of l-NAME-treated offspring *vs.* control at 12 weeks of age (Table 2). Next, there were 8 signaling pathway identified as the significant KEGG pathways in the kidney of offspring to maternal l-NAME + CIT therapy (Table 2). We found that four out of the 140 DEGs, namely *Guca2b*, *Hmox1*, *Hba2*, and *Hba-a2*, were related to regulation of BP (GO: 0008217); three of these genes were related to response to oxidative stress (GO: 0006979), including *Dusp1*, *Hmox1*, and *Serpine1*. We also observed three significantly related KEGG pathways, including circadian rhythm, prion diseases, and mitogen-activated protein kinases (MAPK) signaling pathway (Table 3).

**Table 2:** Significantly regulated KEGG pathways in the kidney of l-NAME-treated offspring and l-NAME + CIT-treated offspring *vs.* control at three months of age.

Term	Count	%	*p*-Value	Benjamini
l-NAME				
Circadian rhythm	4	1.1	7.3 × 10^−4^	7.2 × 10^−2^
MAPK signaling pathway	12	3.3	1.3 × 10^−3^	6.4 × 10^−2^
Colorectal cancer	5	1.4	2.8 × 10^−2^	6.2 × 10^−1^
Chemokine signaling pathway	7	1.9	3.5 × 10^−2^	6.0 × 10^−1^
Pathways in cancer	10	2.8	3.6 × 10^−2^	5.3 × 10^−1^
Wnt signaling pathway	6	1.7	5.6 × 10^−2^	6.2 × 10^−1^
NOD-like receptor signaling pathway	4	1.1	5.8 × 10^−2^	5.8 × 10^−1^
Renal cell carcinoma	4	1.1	7.6 × 10^−2^	6.3 × 10^−1^
Prion diseases	3	0.8	8.9 × 10^−2^	6.5 × 10^−1^
l-NAME + CIT				
Circadian rhythm	3	1.0	1.3 × 10^−2^	7.0 × 10^−1^
PPAR signaling pathway	5	1.6	1.5 × 10^−2^	5.1 × 10^−1^
Chemokine signaling pathway	7	2.3	2.8 × 10^−2^	5.9 × 10^−1^
Histidine metabolism	3	1.0	4.2 × 10^−2^	6.3 × 10^−1^
Metabolism of xenobiotics by cytochrome P450	4	1.3	4.8 × 10^−2^	6.0 × 10^−1^
Butanoate metabolism	3	1.0	7.3 × 10^−2^	6.9 × 10^−1^
Drug metabolism	4	1.3	7.4 × 10^−2^	6.4 × 10^−1^
Prion diseases	3	1.0	8.1 × 10^−2^	6.3 × 10^−1^

**Table 3:** Significantly regulated KEGG pathways in the kidney shared by l-NAME and l-NAME + CIT-treated offspring *vs.* control at three months of age.

KEGG Pathway	Count	Gene Symbol	*p*-Value	Benjamini
Circadian rhythm	3	*Arntl*, *Nr1d1*, *Per3*	3.2 × 10^−3^	1.7 × 10^−1^
Prion diseases	3	*Ccl5*, *Egr1*, *Hspa1a*	2.2 × 10^−2^	4.8 × 10^−1^
MAPK signaling pathway	5	*Cacna1h*, *Dusp1*, *Dusp6*, *Gadd45g*, *Hspa1a*	9.7 × 10^−2^	8.6 × 10^−1^

### 2.3. The Effects of l-NAME and CIT on Gene Expression of Epigenetic Regulators

Epigenetic regulation is a mechanism proposed to contribute to nutrition-induced developmental programming [14]. We therefore investigated five groups of epigenetic regulators, including DNA methyltransferases, histone deacetylases, histone methyl- and acetyl-transferase, bromodomain-containing proteins recognizing acetylated lysine residues, and chromodomain-containing proteins recognizes methylated histones. We identified two genes above the chosen threshold, namely *Dnmt3l* and *Hdac9* (Table 4).

**Table 4:** Changes in genes involved in epigenetic regulation in the kidney of offspring at three months of age to maternal l-NAME and/or CIT *vs.* control.

Gene ID	Gene Symbol	Description	Fold Changes l-NAME/Control	Fold Changes l-NAME + CIT/Control
**DNA methyltransferases**				
ENSRNOG00000039859	*Dnmt1*	DNA methyltransferase 1	1.01	1.04
ENSRNOG00000026132	*Dnmt2*	DNA methyltransferase 2	0.97	0.96
ENSRNOG00000026649	*Dnmt3a*	DNA methyltransferase 3A	0.74	0.87
ENSRNOG00000010625	*Dnmt3b*	DNA methyltransferase 3B	0.78	0.90
ENSRNOG00000001212	*Dnmt3l*	DNA methyltransferase 3-like	0.61	**0.40**
**Histone deacetylases**				
ENSRNOG00000009568	*Hdac1*	Histone deacetylase 1	0.89	1.00
ENSRNOG00000000604	*Hdac2*	Histone deacetylase 2	1.04	1.13
ENSRNOG00000019618	*Hdac3*	Histone deacetylase 3	0.89	0.99
ENSRNOG00000020372	*Hdac4*	Histone deacetylase 4	0.87	1.21
ENSRNOG00000020905	*Hdac5*	Histone deacetylase 5	1.13	1.07
ENSRNOG00000006791	*Hdac6*	Histone deacetylase 6	0.92	0.89
ENSRNOG00000008308	*Hdac7*	Histone deacetylase 7	1.04	1.03
ENSRNOG00000003122	*Hdac8*	Histone deacetylase 8	1.07	1.01
ENSRNOG00000004158	*Hdac9*	Histone deacetylase 9	**0.46**	ND
ENSRNOG00000031915	*Hdac10*	Histone deacetylase 10	0.96	1.17
ENSRNOG00000006824	*Hdac11*	Histone deacetylase 11	0.85	0.76
**Chromodomain-containing proteins**				
ENSRNOG00000014434	*Chd1*	Chromodomain helicase DNA binding protein 1	0.98	1.22
ENSRNOG00000012716	*Chd2*	Chromodomain helicase DNA binding protein 2	1.11	1.17
ENSRNOG00000009722	*Chd3*	Chromodomain helicase DNA binding protein 3	1.01	0.97
ENSRNOG00000018309	*Chd4*	Chromodomain helicase DNA binding protein 4	0.89	1.24
ENSRNOG00000011268	*Chd5*	Chromodomain helicase DNA binding protein 5	0.77	0.89
ENSRNOG00000025011	*Chd8*	Chromodomain helicase DNA binding protein 6	0.94	1.10
**Bromodomain-containing proteins**				
ENSRNOG00000004538	*Brd1*	Bromodomain containing 1	0.93	1.16
ENSRNOG00000000461	*Brd2*	Bromodomain containing 2	0.75	0.95
ENSRNOG00000006770	*Brd4*	Bromodomain containing 4	0.61	0.94
ENSRNOG00000014419	*Brd7*	Bromodomain containing 7	1.01	1.15
ENSRNOG00000020340	*Brd8*	Bromodomain containing 8	1.12	1.08
ENSRNOG00000015676	*Brd9*	Bromodomain containing 9	0.96	1.05
ENSRNOG00000028641	*Brpf3*	Bromodomain and PHD finger containing 3	0.95	0.58
ENSRNOG00000001453	*Baz1b*	Bromodomain adjacent to zinc finger domain, 1B	1.08	1.25
ENSRNOG00000028816	*Baz2a*	Bromodomain adjacent to zinc finger domain, 2A	0.8	1.14
ENSRNOG00000025148	*Baz2b*	Bromodomain adjacent to zinc finger domain, 2B	0.88	1.23
ENSRNOG00000002073	*Brdt*	Bromodomain, testis-specific	0.64	1.3
ENSRNOG00000001632	*Brwd1*	Bromodomain and WD repeat domain containing 1	0.98	1.23
ENSRNOG00000002291	*Brwd3*	Bromodomain and WD repeat domain containing 3	1.11	1.20
**Histone methyl- and acetyl-transferase**				
ENSRNOG00000019585	*Myst1*	K (lysine) acetyltransferase 8	0.80	0.71
ENSRNOG00000022664	*Myst2*	K (lysine) acetyltransferase 7	1.07	1.32
ENSRNOG00000025174	*Myst3*	K(lysine) acetyltransferase 6A	0.92	1.20
ENSRNOG00000007242	*Ehmt1*	Euchromatic histone-lysine *N*-methyltransferase 1	0.95	1.12
ENSRNOG00000030630	*Ehmt2*	Euchromatic histone-lysine *N*-methyltransferase 2	0.84	0.79
ENSRNOG00000001524	*Hat1*	Histone acetyltransferase 1	1.35	1.14

Quantification for gene expression was calculated as reads per kilobase of exon per million mapped reads (RPKM). Genes that changed by RPKM >0.3 and ≥2-fold differences between l-NAME-treated offspring and/or l-citrulline-treated *vs.* control. Significant results are highlighted in bold. ND = not detectable.

## 3. Discussion

This study provides insight into the interactions between genes and the l-citrulline-NO pathway during reproduction with a particular emphasis on programmed hypertension in the offspring kidney. The main novel findings in this study are: (1) NO inhibition by l-NAME during pregnancy induces programmed hypertension in male offspring at 12 weeks of age, which maternal l-citrulline supplementation prevents; (2) we observed that 383 and 337 genes displayed long-term alterations in response to maternal l-NAME and l-NAME + CIT therapy respectively, in 12-week-old kidney; (3) the identified DEGs in l-NAME and l-NAME + CIT-treated offspring, including *Guca2b*, *Hmox1*, *Hba2* and *Hba-a2*, are related to regulation of BP; and (4) there were three significantly related KEGG pathways in response to l-NAME and CIT exposure, including circadian rhythm, prion diseases, and MAPK signaling pathway.

It has been shown that chronic inhibition of NOS with l-NAME results in hypertension and preeclampsia in pregnant rodents [15,16]. As l-NAME cannot penetrate the placenta, the programming effects of l-NAME on the fetus seem to be driven by impaired uteroplacental perfusion and intrauretrine growth retardation (IUGR) [17]. Although our study lacked the measure of birth weight, previous studies have reported that the offspring of l-NAME-treated pregnant rats exhibited IUGR [16,17]. In line with previous studies [9,10,11,12], our results indicate that early l-citrulline therapy prevents programmed hypertension in adult male offspring exposed to maternal NO deficiency. Some particular candidate genes and pathways related to the programmed hypertension have been studied, including those related to glucocorticoid effects, oxidative stress, epigenetic regulation, alterations of renin-angiotensin system (RAS), impaired tubular sodium handling, and reduction in nephron numbers [2,3,13]. We recently observed that maternal l-citrulline therapy prevented l-NAME-induced programmed hypertension, which was associated with decreased asymmetric dimethylarginine (ADMA, an endogenous NOS inhibitor) level and increased l-arginine-to-ADMA ratio in the kidney, increased urinary cGMP levels, and decreased renal protein level of type 3 sodium hydrogen exchanger (NHE3) [4]. However, currently available data does not indicate a common underlying pathway from different programming models. In an attempt to elucidate the impact of other candidate genes and pathways on NO deficiency-induced renal programming, we used RNA-Seq to analyze these specific groups of genes.

In the RNA-Seq dataset, the identification of four DEGs related to regulation of BP, namely *Guca2b*, *Hmox1*, *Hba2*, and *Hba-a2*, is of particular interest for elucidating possible mechanisms by which l-NAME and/or l-citrulline medicates programmed hypertension. The *Guca2b* gene encodes the guanylate cyclase activator 2B, which is responsive for NO-mediated cGMP production. It is well known that activation of cGMP synthesis leads to vasodilation and BP control. *Hmox1* gene encodes for heme oxygenase 1; and it is critical for maintaining NO/oxidative stress balance and is associated with hypertension [18]. *Hba2* and *Hba-a2* encode for hemoglobin alpha 2 chain and adult chain 2, which are involved in negative regulation of BP [19]. Three of these DEGs are related to oxidative stress—*Dusp1*, *Hmox1*, *and Serpine1*. The *Dusp1* gene encodes mitogen-activated protein kinase/dual-specificity phosphatase 1 (MKP-1/DUSP1). MKP-1 is involved in angiotensin II-induced hypertension [20]. The *Serpine1* gene encodes a member of the serine proteinase inhibitor (serpin) superfamily; given that this member is the principal inhibitor of tissue plasminogen activator and that plasminogen activator inhibitor-1 antagonist was reported to attenuate l-NAME-induced hypertension [21], it is possible that maternal NO deficiency regulates *Serpine1* to elicit programmed hypertension. However, additional studies are required to clarify whether these gene-nutrient interactions are potential targets involved in l-NAME-induced programmed hypertension.

Another observation is that l-NAME and l-NAME + CIT both significantly regulated several KEGG pathways and shared three of them in the kidney, including circadian rhythm, prion diseases, and MAPK signaling pathway. Given the pleiotropic bioactivities of NO that regulate a variety of physiological functions, it is not surprising that several important biological pathways are regulated by l-NAME during nephrogenesis.

Circadian rhythm plays a key role in the control of BP and kidney function [22]. Growing evidence indicates the interrelations between nutrients, circadian clock, and epigenomic programming [23,24]. The current study showed l-NAME causes a significant increase of genes encoding nuclear receptor subfamily 1, group D, member 1 (*Nr1d1*, FC +3.297) and period circadian protein homolog 3 protein (*Per3*, FC +2.789), and a decrease of gene encoding aryl hydrocarbon receptor nuclear translocator-like (*Arntl*, FC −0.166). Our data are in agreement with a previous study that shows that l-arginine can influence circadian rhythm and circadian period gene expression [25]. Next, three DEGs belonging to the prion diseases KEGG pathway are shared by l-NAME and l-NAME + CIT exposure, namely *Ccl5*, *Egr1*, and *Hspa1a*. *Ccl5* encodes for chemokine (C-C motif) ligand 5, an inflammatory chemokine. A recent report showed that *Ccl5* can interact with the NO pathway in angiotensin II-induced vascular hypertension [26]; our data suggest its potential role on programmed hypertension. *Egr1* encodes for early growth response-1, a zinc-finger transcription factor that can be activated by oxidative stress to promote atherosclerosis, diabetes, and pulmonary hypertension [27]. We and others have demonstrated that oxidative stress is involved in the development of hypertension in various programming models [4,7,10,11,12,13]. Thus it is likely that *Egr1* is related to oxidative stress-induced programmed hypertension. *Hspa1a* encodes for heat shock 70 kDa protein 1 (Hsp70). We observed that l-NAME causes a significant decrease of *Hspa1a* (FC −0.24). Since Hsp70 has been found to exhibit atheroprotective effects [28], presumably l-NAME blunts its protective effect on programmed hypertension. Last, it is well known that oxidative stress can induce activation of the MAPK pathway, leading to NO-redox imbalance and development of hypertension [29]. However, whether l-citrulline can restore redox balance by regulation of MAPK pathway to prevent programmed hypertension awaits further elucidation.

So far, few studies have investigated the effects of the l-arginine-NO pathway on the developing epigenome, especially in the kidney. NO might modify the fetal epigenome by direct action or indirect regulation in the expression of epigenetic regulator genes. Despite that more than 350 genes were regulated by l-NAME and/or l-citrulline, only histone deacetylase 9 (*Hdac9*) and DNA (cytosine-5)-methyl-transferase 3-like (*Dnmt3l*) were downregulated by l-NAME and l-NAME + CIT respectively. Our data are in agreement with a previous study that showed that all epigenetic regulator genes are unchanged in the fetal kidney in an IUGR model [30]. These findings suggest that epigenetic regulators might not be the major route by which l-NAME and l-citrulline influence renal programming.

However, it is possible that l-NAME might cause IUGR, and thatour NGS results might be a secondary phenomenon. We cannot absolutely rule out the possibility that epigenetic regulation occurs early during nephrogenesis and thus their influences disappear in later life (*i.e.*, three-months-old). The implications of epigenetic regulation deserves further clarification at an early stage of programming (e.g., one-day-old). Another limitation is that we did not examine gender difference in response to l-NAME exposure in this study. The reason is that cardiovascular events occurred at a later age in females than males; only male offspring were recruited in this study since we detected BP in young adulthood. However, we recognize the importance of gender in developmental programming of hypertension which awaits further elucidation.

## 4. Experimental Section

### 4.1. Animals and Experimental Design

This experiment was approved and performed under the Guidelines for Animal Experiments of Chang Gung Memorial Hospital and Chang Gung University. Virgin Sprague Dawley (SD) rats (10 week-old) were obtained (BioLASCO Taiwan Co., Ltd., Taipei, Taiwan) and maintained in a facility accredited by the Association for Assessment and Accreditation of Laboratory Animal Care International. Male rats were caged with individual females until mating was confirmed. Pregnancy rats received l-NAME administration at 60 mg/kg per day by a subcutaneous osmotic pump (Alza Corporation, Palo Alto, CA, USA) during the whole period of pregnancy [4]. Pregnant rats received continuous infusion of iso-osmotic saline were used as controls. Half of the l-NAME treated rats received 0.25% l-citrulline (Sigma, St. Louis, MO, USA) solution dissolved in drinking water during the entire pregnancy and lactation. After birth, the subjects came from litters were culled to 8 pups to standardize the received quantity of milk and maternal pup care. Each litter was left with the mother until weaning; pups were not weighed at birth to prevent maternal rejection. After weaning, only male offspring were assigned to three groups (*n* = 6/group): control, l-NAME, and l-NAME + citrulline (l-NAME + CIT). Blood pressure (BP) was measured in conscious rats by an indirect tail-cuff method (BP-2000, Visitech Systems, Inc., Apex, NC, USA) [10]. Male offspring were sacrificed at 12 weeks of age. Heparinized blood samples were collected at sacrifice. Kidneys were harvested after perfusion with phosphate buffered saline (PBS), decapsulated, divided into cortex and medulla, and stored at −80 °C for further analysis.

### 4.2. Next-Generation Sequencing and Analysis

Kidneys of control, l-NAME, and l-NAME + CIT group were isolated and snap-frozen for whole-genome RNA next-generation sequencing (RNA-Seq), performed by Welgene Biotech Co., Ltd. (Taipei, Taiwan). Purified RNA was quantified at 260 nm (OD600) by using a ND-1000 spectrophotometer (Nanodrop Technology, Wilmington, DE, USA) and analyzed using a Bioanalyzer 2100 (Agilent Technologies, Santa Clara, CA, USA) with RNA 6000 LabChip kit (Agilent Technologies). All procedures were performed according to the Illumina protocol. For all samples, library construction was performed using the TruSeq RNA Sample Prep Kit v2 for ~160 bp (single-end) sequencing and the Solexa platform (Illumina Inc., San Diego, CA, USA). The sequence was directly determined by sequencing-by-synthesis technology using the TruSeq SBS Kit. Raw sequences were obtained using the Illumina GA Pipeline software CASAVA v1.8, which was expected to generate 30 million reads per sample. Quantification for gene expression was calculated as reads per kilobase of exon per million mapped reads (RPKM). Cufflink v 2.1.1 and CummeRbund v 2.0.0 were used to perform statistical analyses of the gene expression profiles. The output files were further annotated by adding gene functional descriptions and Gene Ontology (GO) classifications. The reference genome and gene annotations were retrieved from the Ensembl database [31]. GO term enrichment and fold enrichment or depletion for gene lists of significantly up- and downregulated genes in kidney were determined. GO analysis for significant genes was performed using KEGG [32] and NIH DAVID Bioinformatics Resources 6.7 [33] to identify regulated biological themes.

### 4.3. Quantitative Real-Time Polymerase Chain Reaction (qPCR)

RNA was extracted using TRIzol reagent treated with DNase I (Ambion, Austin, TX, USA) to remove DNA contamination, and reverse transcribed with random primers (Invitrogen, Carlsbad, CA, USA). The complementary DNA (cDNA) product was synthesis using a MMLV Reverse Transcriptase (Invitrogen). Two-step quantitative real-time PCR was conducted using the QuantiTect SYBR Green PCR Kit (Qiagen, Valencia, CA, USA) and the iCycler iQ Multi-color Real-Time PCR Detection System (Bio-Rad, Hercules, CA, USA). Primers were designed using GeneTool Software (BioTools, Edmonton, AB, Canada) (Table 5). We used 18S rRNA (*R18s*) as a reference. Primer efficiency between 1.8 and 2.2 was acceptable. All samples were run in duplicate. To quantify the relative gene expression, the comparative threshold cycle (*C*_t_) method was employed. For each sample, the average *C*_t_ value was subtracted from the corresponding average r18S value, calculating the Δ*C*_t_. ΔΔ*C*_t_ was calculated by subtracting the average control Δ*C*_t_ value from the average experimental Δ*C*_t_. The fold-increase of the experimental sample relative to the control was calculated using the formula 2^−ΔΔ*C*t^.

**Table 5:** qPCR primers sequences.

Gene	Forward	Reverse
*Bcl6*	5'-CTGAGGGAAGGCAACATCAT-3'	5'-CGGCTGTTCAGGAACTCTTC-3'
*Egr1*	5'-CAGGAGTGATGAACGCAAGA-3'	5'-AGCCCGGAGAGGAGTAAGAG-3'
*Olr1668*	5'-ACGTGGCTATCTGCAGACCT-3'	5'-CTCCCCACAGGCAGTTTTTA-3'
*Sgk1*	5'-GGGCTGTCTTGTATGAGATGC-3'	5'-GTGCCTTGCTGAGTTGGAG-3'
*Dmrtc1c*	5'-ACATACAAGTCACGCTGGCA-3'	5'-TTGGCCTGTTTGAGGGGTTT-3'
*R18s*	5'-GCCGCGGTAATTCCAGCTCCA-3'	5'-CCCGCCCGCTCCCAAGATC-3'

*Bcl6* = B-cell CLL/lymphoma 6; *Egr1* = early growth response-1; *Olr1668* = olfactory receptor 1668; *Sgk1* = serum and glucocorticoid-regulated kinase 1; *Dmrtc1c* = DMRT-like family C1c1.

### 4.4. Statistical Analysis

Data were represented as mean ± S.E.M. For most parameters, statistical analysis was done using 1-way ANOVA with Tukey’s *post hoc* test for multiple comparisons. Blood pressure (BP) was analyzed by 2-way repeated-measures ANOVA and Tukey’s *post hoc* test. A *p*-value <0.05 was considered statistically significant. All analyses were performed using the Statistical Package for the Social Sciences (SPSS) software (IBM, Armonk, NY, USA).

## 5. Conclusions

In conclusion, the impact of nutrition-gene interactions on fetal programming is supported by our results showing that maternal l-citrulline supplementation prevents NO depletion-induced programmed hypertension in adult offspring. Using NGS technology, we identified >350 genes that exhibit long-term alteration in expression levels related to a common phenotype that follows programming insults of l-NAME and/or l-citrulline supplementation. Our NGS results are of significance for the development of potential interventions in the prevention of programmed hypertension in children exposed to maternal NO deficiency.
